# Coexisting metabolic dysfunction-associated steatotic liver disease exacerbates in-hospital outcomes in patients with heat stroke

**DOI:** 10.3389/fmed.2024.1451133

**Published:** 2024-11-12

**Authors:** Ping Zhang, Guo Tang, Hongguang Gao, Tianshan Zhang, Sha Yang, Tao Cheng, Rong Yao

**Affiliations:** Emergency Department of West China Hospital, Sichuan University, Chengdu, Sichuan, China

**Keywords:** MASLD, heat stroke, in-hospital mortality, organ injury, emergency department

## Abstract

**Purpose:**

This study aimed to investigate the impact of coexisting metabolic dysfunction-associated steatotic liver disease (MASLD) on in-hospital mortality and organ injury markers in patients with heat stroke (HS).

**Approach:**

HS patients were retrospectively identified between July 1, 2022 and September 30, 2023 at West China Hospital, Sichuan University. Baseline characteristics, such as demographics, initial vital signs, and organ functional indicators were collected. Outcome events included organ injury and in-hospital mortality. The Least Absolute Shrinkage and Selection Operator (Lasso) method was employed to identify the optimal predictors for in-hospital mortality in HS patients. Subsequently, multivariable logistic regression analysis was performed to assess the relationship between the presence of MASLD and in-hospital mortality as well as organ function indicators.

**Findings:**

A total of 112 patients were included in the study, in which 27 (24.1%) had coexisting MASLD. Compared to those without MASLD, patients with MASLD had higher levels of various organ injury markers such as aspartate aminotransferase, urea nitrogen, serum cystatin C, creatinine, uric acid, myoglobin, creatine kinase and its isoenzymes upon admission (*P* < 0.05). The multivariable Logistic regression analysis indicated that the presence of MASLD is an independent risk factor for in-hospital mortality in HS patients.

**Conclusion:**

This study firstly indicated that coexisting MASLD may exacerbate organ injury in HS patients and serve as an independent risk factor for in-hospital mortality.

## 1 Introduction

As global temperatures rise, heat-related illnesses (HRI) are gaining more attention (1). Heat stroke (HS) is the most severe form of HRI, characterized by high fever, altered consciousness levels, and dysfunction of multiple organs, leading to a life-threatening condition (2, 3). The liver is significantly impacted in HS, with severe hepatic injury often contributing directly to patient mortality. Various factors, including direct heat injury, reduced blood flow to the viscera, and the development of systemic inflammatory response syndrome (SIRS), can contribute to liver injury in HS (4, 5). The systemic immune response to HS has been linked to intestinal cellular dysfunction, causing the gastrointestinal tract to become leaky, allowing endotoxins to enter the bloodstream. With liver injury, the liver’s ability to clear gut-derived endotoxins weakened, further increasing endotoxin concentrations in the blood, leading to tissue damage and more severe inflammation. Moreover, the translocation of bacteria and endotoxins can activate the liver’s defense mechanisms, particularly Kupffer cells, initiating a series of inflammatory responses and the release of substantial cytokines, which can lead to SIRS and further exacerbate multi-organ injury (6, 7). Ultimately, the liver plays a crucial role in the pathogenesis of HS and contributes significantly to the progression of the disease (8).

Excessive oxidative stress in liver cells is a major factor in the abnormal death of hepatocytes and liver injury caused by HS (9). The liver, being the primary organ for fat synthesis, contains high levels of oxygen and lipids, making it particularly vulnerable to oxidative stress and lipid peroxidative damage (10). A key question arises: does fatty fill in the whole liver, as seen in metabolic dysfunction-associated steatotic liver disease (MASLD) or previously known as non-alcoholic fatty liver disease (NAFLD), increase its susceptibility to heat-induced injury? (11).

MASLD is the most prevalent chronic liver condition, affecting approximately 26–30% in Asian health checkup population (12). It’s common for individuals to suffer from metabolic diseases. Despite this, there is a lack of published research examining the influence of concurrent MASLD on the prognosis of HS. This study aims to explore the impact of MASLD on organ dysfunction and prognostic outcomes in HS patients, with the goal of enhancing treatment strategies and ultimately improving clinical outcomes for these patients.

## 2 Materials and methods

### 2.1 Study design and participants

This research is a retrospective observational analysis, enrolling adult HS patients admitted to the emergency department (ED) of West China Hospital, Sichuan University between July 1, 2022, and September 30, 2023. The diagnosis of HS based primarily on the triad of hyperthermia, neurologic abnormalities, and recent exposure to hot weather (in the classic form) or physical exertion (in the exertional form). tachypnea, and hypotension are common (13). Exclusion criteria include: (1) age less than 18 years; (2) incomplete or missing medical histories; (3) a history of long-term alcohol use; (4) concurrent chronic liver disease other than fatty liver; (5) other etiologies causing liver injury, such as infectious, pharmacological, or toxic; (6) lack of abdominal imaging studies (ultrasound, CT).

The primary outcomes in-hospital mortality was defined as death of a patient that occurs during their stay in the hospital. Regarding relative indicators, the proposed criteria for a positive diagnosis of MASLD are based on histological (biopsy), imaging or blood biomarker evidence of fat accumulation in the liver (hepatic steatosis) in addition to one of the following three criteria, namely overweight/obesity, presence of type 2 diabetes mellitus (T2DM), or evidence of metabolic dysregulation (14). We take the imaging criteria in this study. Kidney injury was assessed using the KDIGO (Kidney Disease: Improving Global Outcomes) criteria, which rely on serum creatinine levels and urine output (15). The diagnosis of acute liver injury (ALI) is primarily based on liver function tests, with a focus on serum alanine aminotransferase (ALT) levels. A key diagnostic criterion is a marked elevation in ALT, typically exceeding 5–10 times the upper limit of normal, indicating significant hepatocellular damage (16, 17). Our study uses the ESC Clinical Practice Guidelines, with the primary basis for diagnosing myocardial injury being elevated cardiac troponin levels (18).

Outcomes in the study were measured using standardized protocols and objective clinical criteria, including pre-specified cutoffs for laboratory values and diagnostic tools such as imaging and scoring systems. To minimize bias, outcome assessors were blinded to the study groups, and independent reviews were conducted when necessary. In cases where subjective interpretation was possible, predefined scoring systems (e.g., SOFA score) were used, and all cases were adjudicated by multiple blinded assessors to ensure objectivity and consistency.

The research strictly adhered to the ethical guidelines for human medical research outlined in the Declaration of Helsinki. Since the study was retrospective and patient identifiers were anonymized during data collection, participant consent was not necessary. Approval for the project was obtained from the Ethical Committee of West China Hospital, Sichuan University (Ethical Approval No.: 2022-1591). The research also followed the Transparent Reporting of a multivariable prediction model for Individual Prognosis or Diagnosis (TRIPAD) guidelines (19).

### 2.2 Data collection

Patient demographic and clinical data upon admission were retrospectively retrieved from the electronic medical records, comprising gender, age, HS classification, pulse, respiratory rate, systolic and diastolic blood pressures, total bilirubin, alanine aminotransferase, aspartate aminotransferase, blood urea nitrogen, serum creatinine, cystatin C, uric acid, creatine kinase, creatine kinase isoenzymes, myoglobin, troponin, prothrombin time, international normalized ratio, activated partial thromboplastin time (APTT), thrombin time, fibrinogen, D-dimer, Sequential Organ Failure Assessment (SOFA) score, Glasgow Coma Scale (GCS) score were calculated for all patients on admission. The main results, survival condition at discharge were analyzed.

### 2.3 Statistical analysis methods

Statistical analysis was performed using R 4.3.1 software. For missing data, multiple imputation was applied using the “mice” package in R, ensuring that missing values were filled by generating multiple datasets, each with different plausible values. These imputed datasets were then pooled for subsequent analyses to account for uncertainty due to missing data. Outcomes that obey normal distribution are expressed as (x ± s), and the *t*-test is used for comparison between groups. Skewed distribution data were expressed as M (QR), and the Mann-Whitney U test was used for comparison between groups. Count data were expressed as n (%), and comparisons between groups were performed using the chi-square test or Fisher’s exact probability method. Least absolute shrinkage and selection operator (Lasso) regression was used to screen the best predictor variables of in-hospital death in HS patients, followed by multivariate logistic regression analysis to evaluate the correlation between combined MASLD and in-hospital death in HS patients. Additionally, the correlation between MASLD and organ function indicators in patients with HS was explored.

## 3 Results

### 3.1 Baseline characteristics

A total of 112 patients were included in this study, with 27 (24.1%) diagnosed with MASLD. Pulse rate in Patients with MASLD were higher than those in surviving non-MASLD patients (*P* < 0.05). No significant differences were observed in other vital signs, proportion of exertional HS, gender, age, or other indicators between the two groups at the time of consultation (Table 1).

**TABLE 1:** Comparison of initial clinical characteristics of HS patients with and without MASLD at admission.

Variable	Non-MASLD group (*N* = 85)	MASLD group (*N* = 27)	*P-*value
Exertion-induced HS (%)	43 (50.6%)	17 (63.0%)	0.367
Gender (male) (%)	51 (60.0%)	19 (70.4%)	0.458
Age (years)	57 [42.0, 70.0]	65 [50.5, 74.5]	0.290
Temperature (°C)	37.9 [36.8, 39.0]	38.1 [37.1, 39.5]	0.310
Heart rate (beat/min)	98 [85, 113]	108 [93, 132]	0.049
Respiratory rate (beats/min)	20 [20, 23]	22 [20, 30]	0.149
Systolic blood pressure (mmHg)	123 [106, 140]	116 [100, 132]	0.324
Diastolic blood pressure (mmHg)	73 [64, 86]	65 [56, 79]	0.157
Peripheral oxygen saturation (%)	97 [95, 99]	96 [95, 99]	0.954

HS, heat stroke; MASLD, metabolic dysfunction-associated steatotic liver disease.

### 3.2 Organ injury and in-hospital mortality

Compared with patient in non-MASLD group, patients with MASLD had higher levels of aspartate aminotransferase, urea nitrogen, serum cystatin C, creatinine, uric acid, myoglobin, creatine kinase (*P* < 0.05). However, there were no significant differences in total bilirubin, troponin, platelet count, coagulation-related indexes, and fibrinogen-related indexes between the two groups. Additionally, the in-hospital mortality rate was significantly higher in the MASLD group compared to the non- MASLD group (10% vs 0%, *P* < 0.001) (Table 2).

**TABLE 2:** Comparison of organ dysfunction markers and in-hospital mortality between MASLD and non-MASLD HS patients.

Variable	Non-MASLD group (*N* = 85)	MASLD group (*N* = 27)	*P-*value
Total bilirubin (μmol/l)	21.3 [13.9, 36.2]	22.5 [15.3, 34.5]	0.598
Alanine aminotransferase (IU/L)	68 [39, 155]	125 [57, 342]	0.137
Aspartate aminotransferase (IU/L)	96 [48, 249]	216 [71.5, 860]	0.049
Urea nitrogen (mmol/L)	7.50 [5.40, 11.2]	12.4 [7.40, 17.6]	0.013
Cystatin C (mg/L)	1.11 [0.93, 1.52]	1.81 [1.09, 2.57]	0.006
Serum creatinine (μmol/L)	86 [68, 153]	183 [85, 270]	0.014
Uric acid (mmol/L)	287 [185, 378]	436 [277, 598]	0.004
Myoglobin (ng/ml)	681 [162, 1859]	2267 [411, 3000]	0.028
Creatine kinase (ng/ml)	768 [274, 2830]	2097 [555, 5836]	0.049
Creatine kinase-MB (ng/ml)	9.31 [3.91, 26.10]	25.6 [5.50, 85.60]	0.047
Troponin I (ng/L)	68.6 [22, 381]	112 [27.2, 246]	0.716
Platelet count (10^9/L)	93 [42.0, 143]	63 [29.5, 118]	0.134
Prothrombin time (s)	12.5 [11.5, 15.7]	14.1 [11.9, 23.4]	0.099
International normalized ratio	1.16 [1.06, 1.42]	1.38 [1.10, 2.17]	0.054
APTT (s)	30.9 [27.7, 36.1]	28.2 [26.9, 53.0]	0.688
Thrombin time (s)	17.8 [16.6, 20.1]	18.4 [17.4, 30.6]	0.093
Fibrinogen (g/L)	2.69 [1.79, 3.60]	2.11 [1.20, 3.23]	0.194
Fibrinogen degradation products (mg/L)	12.0 [5.70, 23.5]	20.7 [7.15, 30.0]	0.190
D-Dimer (mg/L FEU)	4.47 [2.02, 13.3]	8.00 [3.20, 15.1]	0.198
Outcome (death) (%)	0 (0%)	10 (37.0%)	<0.001

HS, heat stroke; MASLD, metabolic dysfunction-associated steatotic liver disease; APTT, activated partial thromboplastin time.

### 3.3 Prognostic factors of mortality

10 patients (9%) passed away in hospital, all of whom belonged to the MASLD group. The in-hospital mortality rate for patients with MASLD was notably higher compared to those without MASLD (10% vs. 0%, *P* < 0.001). In the non-survivor group, patients had significantly higher proportions of MASLD, pulse at presentation, SOFA score, urea nitrogen, serum cystatin C, creatinine, uric acid, myoglobin, creatine kinase, fibrinogen degradation products, and D-dimer compared to those in the surviving group (*P* < 0.05) (Table 3).

**TABLE 3:** Comparison of baseline characteristics between in-hospital mortality and survival groups in HS patients.

Variable	Surviving group (*N* = 102)	Non-survivor group (*N* = 10)	*P-*value
Prevalence of MASLD comorbidity (%)	17 (16.7%)	10 (100%)	<0.001
Exertion-induced HS (%)	55 (53.9%)	5 (50.0%)	1.000
Gender (male) (%)	63 (61.8%)	7 (70.0%)	0.741
Age (years)	57.5 [44.5, 69.8]	73.5 [59.5, 74.8]	0.183
Temperature (°C)	38.0 [36.8, 39.0]	37.6 [36.7, 39.4]	0.951
Heart rate (beat/min)	99 [86, 113]	129 [103, 140]	0.040
Respiratory rate (beats/min)	21 [20, 24]	21 [18.5, 28]	0.963
Systolic blood pressure (mmHg)	124 [105, 139]	115 [100, 134]	0.551
Diastolic blood pressure (mmHg)	72.5 [60.5, 83.0]	62.5 [54.5, 78.8]	0.337
Peripheral oxygen saturation (%)	97.0 [95.0, 99.0]	96 [92.8, 96.8]	0.299
GCS score	6.00 [3.00, 13.00]	4.00 [3.00, 5.75]	0.058
SOFA score	7.00 [4.00, 10.00]	9.50 [8.25, 12.50]	0.030
Total bilirubin (μmol/l)	15.9 [12.0, 25.9]	18.0 [16.7, 19.8]	0.717
Alanine aminotransferase (IU/L)	52.0 [23.0, 86.8]	94.0 [39.5, 200.0]	0.175
Aspartate aminotransferase (IU/L)	67.5 [36.0, 164.0]	218 [48.2, 455.0]	0.105
Urea nitrogen (mmol/L)	6.50 [4.80, 10.20]	10.40 [8.88, 12.60]	0.012
Cystatin C (mg/L)	1.00 [0.80, 1.41]	1.51 [1.14, 1.79]	0.023
Serum creatinine (μmol/L)	85.5 [65, 152]	185 [138, 252]	0.012
Uric acid (mmol/L)	269 [174, 353]	416 [337, 538]	0.036
Myoglobin (ng/ml)	560 [114, 1943]	3000 [766, 3000]	0.015
Creatine kinase (ng/ml)	512 [153, 2220]	2030 [548, 11481]	0.022
Creatine kinase-MB (ng/ml)	6.28 [1.94, 20.30]	21.9 [2.90, 170.0]	0.209
Troponin I (ng/L)	58.0 [20.6, 225.0]	171 [63.1, 535.0]	0.054
Platelet count (10^9/L)	130 [73.8, 198]	105 [57.5, 160]	0.606
Prothrombin time (s)	12.1 [11.1, 14.2]	14.4 [12.0, 23.0]	0.100
International normalized ratio	1.13 [1.03, 1.32]	1.33 [1.08, 2.15]	0.101
APTT (s)	27.8 [25.6, 32.1]	31.4 [27.7, 53.7]	0.040
Thrombin Time (s)	17.7 [16.4, 19.6]	18.4 [16.5, 36.4]	0.400
Fibrinogen (g/L)	2.81 [1.87, 4.12]	2.09 [1.24, 3.51]	0.268
Fibrinogen degradation products (mg/L)	8.00 [4.53, 18.50]	28.40 [17.50, 53.80]	0.006
D-Dimer (mg/L FEU)	2.55 [1.38, 8.70]	16.30 [6.86, 32.60]	0.004

MASLD, metabolic dysfunction-associated steatotic liver disease; HS, heat stroke; SOFA, Sequential Organ Failure Assessment; GCS, Glasgow Coma Scale; APTT, activated partial thromboplastin time.

**FIGURE 1:**
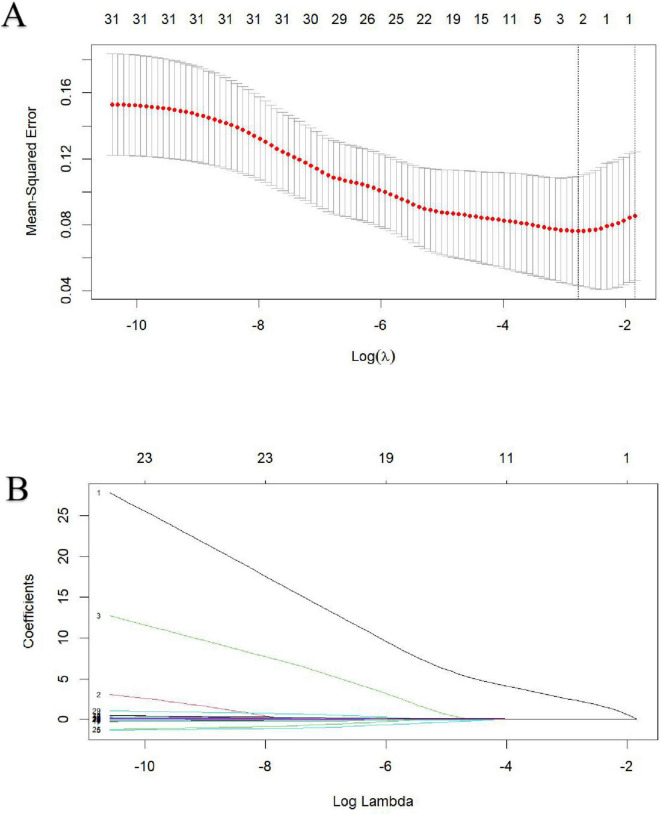
Identification of risk factors for in-hospital mortality in heat stroke patients using Lasso regression with admission indices. **(A)** The best penalty coefficient λ for Lasso regression is ascertained through 10-fold cross-validation and the one standard error (1SE) rule. **(B)** The Lasso regression coefficient plot for clinical features.

Lasso regression was utilized to identify MASLD, APTT and D-dimer levels at admission as risk factors for non-survivor patients with HS (Figures 1A,B). These indicators were subsequently incorporated into a multivariate logistic regression analysis, using the forward stepwise regression method. Finally, two independent risk factors were identified as independent risk factors for in-hospital mortality, including prevalence of MASLD comorbidity and increased D-Dimer levels at admission, while the APTT failed to show significant impact on survival (Table 4).

**TABLE 4:** Multivariable logistic regression analysis for in-hospital mortality risk factors in HS patients.

Variable	crude OR (95% CI)	adj.OR (95% CI)	P (Wald’s test)	P (LR-test)
Prevalence of MASLD comorbidity (%)	502667899.6 (0, Inf)	3953722479.54 (0, Inf)	0.994	<0.001
D-dimer (mg/L FEU)	1.07 (1.02, 1.12)	1.14 (1.02, 1.28)	0.025	0.003
APTT (s)	1.02 (1.00, 1.04)	1.04 (0.92, 1.17)	0.520	0.167

HS, heat stroke; adj.OR, adjusted odds ratio; LR-test, likelihood ratio-test; APTT, activated partial thromboplastin time.

## 4 Discussion

The study firstly reported the potential effects of MASLD comorbidity on the outcomes of HS patients. We discovered significant information in in-hospital mortality, liver function indicators, renal function levels, and muscle enzyme levels between patients with or without MASLD, indicating that the presence of MASLD could intensify organ dysfunction in HS patients. Multivariate logistic regression analysis demonstrated that coexisting MASLD independently increases the risk of in-hospital mortality among HS patients. As the global incidence of HS continues to rise, and MASLD emerges as the most common chronic liver disease, the results of this study offer valuable insights for assessing and treating HS.

Prevalence data from 245 studies, encompassing a total of 2,699,627 individuals, were analyzed using a hierarchical Bayesian model to project the future prevalence of MASLD through 2040. Projections indicate that, by 2040, more than half of the adult population will be affected by MASLD around the world. The global burden of liver fibrosis associated with MASLD is expected to rise two- to three-fold over the next decade (20). Previous studies have shown a strong link between MASLD and metabolic conditions like obesity, insulin resistance, hypertension, and dyslipidemia (21). Liver injury caused by MASLD involves a complex interaction of multiple critical mechanisms. Initially, insulin resistance induces the buildup of lipids in liver cells, resulting in hepatic steatosis. Adipocytes and immune cells release pro-inflammatory cytokines, resulting in long-term chronic inflammation of the liver, steatosis, hepatocyte mitochondrial dysfunction, and increased production of reactive oxygen species (ROS). This oxidative stress and endoplasmic reticulum (ER) stress can contribute to liver disease, causing cell damage and apoptosis (22). MASLD is associated with dysregulated lipid metabolism, which includes increased *de novo* lipogenesis and decreased fatty acid oxidation. This imbalance leads to the accumulation of lipids in the liver and cellular stress. Research indicates that genetic predisposition and epigenetic changes play a role in the development and progression of MASLD (23). The intricate interaction of these mechanisms highlights the progressive aspect of MASLD and underscores the importance of a multifaceted therapeutic approach aimed at various pathways to alleviate liver injury and prevent the advancement of the disease.

The liver is a major target organ for HS, as highlighted by Savioli et al. (8). Their research indicates that the liver not only sustains significant damage during HS, but also plays a crucial role in the development of HS. This study investigating the relationship between HS and MASLD, clinical data from MASLD and non-MASLD groups of HS patients were analyzed. Although no statistical difference was observed, a higher proportion of exertional fever in the MASLD group suggests that these patients may have lower tolerance to physical labor in high-temperature environments. This intolerance may be linked to metabolic disorders and reduced heat tolerance in MASLD patients. Research suggests that MASLD may result in diminished heat tolerance, potentially due to decreased expression of heat shock protein 72 (HSP72) and heightened activation of c-Jun N-terminal kinase (JNK) (24–27). Heat resistance, or the heat shock response, is a protective defense mechanism against thermal stress, characterized by the increased production of heat shock proteins, particularly HSP72 (24). HSP72 exerts its anti-inflammatory effects by inhibiting the activation of JNK, a key negative regulator in insulin signaling (21). In MASLD, the increased activation of JNK leads to inhibitory phosphorylation of insulin receptor substrate 1, resulting in insulin resistance and hyperinsulinemia (26). These metabolic disturbances impact various physiological processes and contribute to the progression of MASLD. Furthermore, JNK can also stimulate the production of reactive oxygen species by inhibiting mitochondrial respiration, which in turn triggers cell apoptosis and mitochondrial dysfunction (27).

The liver, serving as a key metabolic organ, participates in processes such as glucose and lipid metabolism. When HS occurs, metabolic disorders like insulin resistance, elevated blood glucose levels, and lipid abnormalities can worsen the tissue damage induced by heat stress. Thermal shock induces a systemic inflammatory response by prompting the release of pro-inflammatory cytokines like interleukin-6 (IL-6) and tumor necrosis factor-α (TNF-α) from tissues and organs. The liver, being a primary site for cytokine production and immune regulation, is closely linked to the intensity and duration of systemic inflammation. Recognition and response to danger signals such as pathogen-associated molecular patterns (PAMPs) and damage-associated molecular patterns (DAMPs) lead to the production of pro-inflammatory cytokines like IL-6 and TNF-α, which drive systemic inflammatory responses. Over time, multiple inflammatory factors contribute to the gradual progression of systemic inflammation and organ dysfunction in HS (28–32). Some studies suggest that MASLD greatly elevates the risk of cardiovascular disease through the promotion of atherosclerosis, endothelial dysfunction, and inflammatory pathways (33). Additionally, MASLD is linked independently to a higher risk of chronic kidney disease (34). Currently, there is a lack of research on how MASLD impacts the pathophysiological mechanisms of organ function damage in HS patients. Future studies are needed to explore this further, with the potential to enhance early detection and ultimately improve patient outcomes.

In light of the higher prevalence of MASLD comorbidity in this study, baseline characteristics indicated that non-survivors exhibited poorer renal function, heart function, coagulation disorders, and higher heart rates, SOFA scores, and a greater tendency toward disseminated intravascular coagulation (DIC) compared to the surviving group. Increased heart rate and elevated SOFA score upon admission (35), elevated Troponin I levels (36), as well as hepatic dysfunction and renal insufficiency (37, 38) have been consistently linked to poor prognoses; however, robust conclusions remain elusive due to small sample sizes and great heterogeneity among studies. While most variables were excluded by Lasso regression, leaving only MASLD comorbidity, APTT, and D-dimer to be identified as the best predictors, the possibility remained that some meaningful risk factors were unintentionally excluded, potentially due to the small sample size. Furthermore, despite a futile APTT, elevated D-dimer levels was also demonstrated to be significantly associated with in-hospital mortality by a subsequent multivariate logistic regression analysis. Another Chinese study showed that combined APTT and D-dimer levels could serve as reliable biomarkers to predict the severity of HS, providing crucial insights into patient prognosis (39). Elevated D-dimer levels usually linked to multiple organ damage, DIC, and other serious conditions (40), thus exacerbating the risk of death in HS.

The retrospective design of this study introduces potential limitations, such as data incompleteness and inherent biases, which may affect the accuracy of the findings. For example, we did not consider the severity of HS in our study. Meanwhile, the exclusion of patients lacking abdominal imaging or with other forms of chronic liver disease could introduce selection bias, potentially impacting the study’s results. Additionally, the relatively small sample size limits the generalizability and external validity of the findings. The geographic and population-specific nature of the study may also restrict the applicability of its conclusions to broader populations or regions. Furthermore, the analysis was confined to in-hospital outcomes without long-term follow-up for survival and prognosis after discharge, which could influence the overall interpretation of patient outcomes. Lastly, other metabolic dysfunction-associated illnesses like hyperlipidemia and insulin resistance may influence MASLD and in-hospital mortality, but their roles were not fully clarified because of their difficulties in diagnosis when patients were in acute phase of HS. To enhance the robustness of future research, we recommend including larger cohorts in prospective studies and extending the follow-up period to better assess long-term outcomes.

## 5 Conclusion

This study is the first to find that combined MASLD is an independent risk factor for in-hospital death in HS patients and exacerbates organ function damage in these patients. These findings offer a fresh insight into the clinical management of HS and underscore the significance of preventing HS and providing timely treatment for MASLD patients. Future research should delve deeper into the interaction mechanism between MASLD and HS to enhance treatment strategies and lower the mortality rate of HS patients.

## Data Availability

The original contributions presented in this study are included in this article/supplementary material, further inquiries can be directed to the corresponding author.
